# Follicular Lymphoma Tregs Have a Distinct Transcription Profile Impacting Their Migration and Retention in the Malignant Lymph Node

**DOI:** 10.1371/journal.pone.0155347

**Published:** 2016-05-26

**Authors:** Hristina Nedelkovska, Alexander F. Rosenberg, Shannon P. Hilchey, Ollivier Hyrien, W. Richard Burack, Sally A. Quataert, Christina M. Baker, Mitra Azadniv, Stephen L. Welle, Stephen M. Ansell, Minsoo Kim, Steven H. Bernstein

**Affiliations:** 1 James P. Wilmot Cancer Center, Lymphoma Biology Program, Department of Medicine University of Rochester Medical Center, Rochester, New York, United States of America; 2 Division of Allergy, Immunology, and Rheumatology, Department of Medicine, University of Rochester Medical Center, Rochester, New York, United States of America; 3 Department of Biostatistics and Computational Biology, University of Rochester Medical Center, Rochester, New York, United States of America; 4 David H. Smith Center for Vaccine Biology and Immunology, Aab Institute of Biomedical Sciences, Department of Microbiology and Immunology, University of Rochester Medical Center, Rochester New York, United States of America; 5 University of Rochester Genomics Research Center, University of Rochester Medical Center, Rochester, New York, United States of America; 6 Division of Hematology, Mayo Clinic, Rochester, Minnesota, United States of America; Jackson Laboratory, UNITED STATES

## Abstract

We have previously shown that regulatory T cells (Tregs) infiltrating follicular lymphoma lymph nodes are quantitatively and qualitatively different than those infiltrating normal and reactive nodes. To gain insight into how such Treg populations differ, we performed RNA sequence (RNAseq) analyses on flow sorted Tregs from all three sources. We identify several molecules that could contribute to the observed increased suppressive capacity of follicular lymphoma nodal tregs, including upregulation of CTLA-4, IL-10, and GITR, all confirmed by protein expression. In addition, we identify, and confirm functionally, a novel mechanism by which Tregs target to and accumulate within a human tumor microenvironment, through the down regulation of S1PR1, SELL (L-selectin) and CCR7, potentially resulting in greater lymph node retention. In addition we identify and confirm functionally the upregulation of the chemokine receptor CXCR5 as well as the secretion of the chemokines CXCL13 and IL-16 demonstrating the unique ability of the follicular derived Tregs to localize and accumulate within not only the malignant lymph node, but also localize and accumulate within the malignant B cell follicle itself. Such findings offer significant new insights into how follicular lymphoma nodal Tregs may contribute to the biology of follicular lymphoma and identify several novel therapeutic targets.

## Introduction

Follicular lymphoma is the second most common type of non-Hodgkin’s lymphoma and despite its indolent nature, it is essentially incurable.[1] Gene expression and immuno-histochemistry studies suggest that immune microenvironment is a critical determinant of the natural history of this disease showing strong correlation between number, type and anatomical location of immune-effector cells in pre-treatment patient biopsies with treatment outcome.[2–7] Regulatory T cells (Tregs), a CD4^+^ T helper cell population that suppresses both CD4^+^ and CD8^+^ cell priming and effector function, and normal germinal center (GC) B cell proliferation and antibody production has emerged as one such critical immune-effector cell population having prognostic significance.[2, 3, 8, 9] We and others have shown that the proportion of CD4^+^ T-cells infiltrating FL lymph nodes (FL) with a Treg phenotype is greater than that seen in normal lymph nodes (NLN).[10]

FL Tregs potently inhibit FL effector CD4^+^ and CD8^+^ T cell proliferation and cytokine production and appear to have greater suppressor function than that of Tregs infiltrating NLN, as we have shown.[10] The mechanisms of such suppression are mediated partly by TGF-beta, adenosine, and PD-1/PD-1L interactions.[10–14] It would thus be anticipated that FL Tregs would inhibit any endogenous anti-tumor effector T cell responses or those elicited by immunotherapy with or without chemotherapy. If so, increased number of Tregs should be associated with worse outcome as is seen with many, but not all solid tumors, however there are conflicting data regarding prognostic significance of Tregs in FL.[2, 3, 5, 7, 15–18] These potential contradictory effects of Tregs on clinical outcome is likely due to the balance of Tregs in the microenvironment relative to other immune-effector cells, and the type and anatomical distribution of the Tregs dictating their net biological activity.

Tregs have been shown to up-regulate the canonical transcription factor of the specific T-cell populations they suppress in response to unique microenvironmental signals.[8, 19–21] This results in their expression of a chemokine receptor profile that drives these Tregs to traffic to and suppress their target T cell population. For example, Tregs that express T-bet up-regulate CXCR3 driving them to traffic to sites of Th1 inflammation and thus suppress Th1 cells. Similarly, Tregs that localize in the GC are a distinct subset of Tregs called T-follicular regulatory cells (TFR).[8, 22–25] Such cells modulate the B-cell GC response by limiting the number and function of T-follicular helper (TFH) cells. In mice, TFR are derived from naïve Tregs that up-regulate Bcl-6 upon activation, resulting in CXCR5 expression directing such cells to the GC via gradients of CXCL13.[8, 24, 25] We and others have shown an increased proportion of TFR in FL compared to that seen in NLN and in contrast to observations in mice, we have shown that human FL TFR are partially derived from TFH.[26] TFH cells support the viability of FL B-cells.[22] In addition, the TFH-B-cell axis is a critical pathway in the biology of several types of cancer.[27–31] Taken together, Tregs that inhibit tumor specific TH1 cells would be expected to have different effects on tumor growth than Tregs that inhibit TFH cells in tumors, where these cells play a tumor supporting role. Therefore, molecular events that modulate Treg migration and trafficking would be anticipated to partly dictate whether Tregs have a tumor suppressive and/or promoting role.

Although Tregs play a fundamental role in FL biology, significant questions remain regarding how such Tregs infiltrating FL differ from those infiltrating NLN, especially for homing, migration and function. To this end, we performed RNAseq analysis of purified Tregs from malignant FL. As we had the unique ability to obtain NLN, we performed RNAseq on their sorted Tregs. Finally, we also performed RNAseq on Tregs from pathologically enlarged reactive lymph nodes (RLN), biopsies of which showed reactive lymphoid hyperplasia as seen with infectious or inflammatory processes.

The following gene, protein and functional studies resulted in several key findings: 1) many differentially expressed genes in FL vs. NLN are related to cell migration and movement, 2) FL Tregs have the unique ability to localize within the malignant B-cell follicle, in part through an autocrine CXCL13-CXCR5 axis, 3) production and secretion of IL-16 by FL Tregs as well as increased retention of Tregs in the FL resulting from irreversible down-regulation of S1P1 may partly account for observed increased frequency of Tregs in FL relative to NLN, 4) FL Tregs may modulate tumor microenvironment through their elicitation of IL-16 and CCL4, and 5) Several molecules, including CTLA-4, IL-10 and GITR may comprise a pattern that could account for the increased suppressive capacity of FL Tregs.

## Methods

### Patient Samples

Primary FL and RLN were obtained from, 12 and 5 patients respectively, undergoing routine biopsy (Table D in S1 File). NLN were obtained from 10 different patients undergoing vascular surgery where obstructive LNs are removed. Single cell suspensions (SCSs) were prepared as previously described.[10, 32] Primary samples were acquired under a University of Rochester Institutional Review Board approved protocol and written consent was obtained.

### Flow Cytometry and Cell Sorting

Cryopreserved SCSs were thawed (one freeze/thaw cycle), washed once in FACS buffer (PBS, 1% heat-inactivated FBS), stained with surface antibodies, washed with FACS buffer. Stained cells were fix/permeabilzed and stained with intracellular antibodies using the human Treg (FoxP3) staining kit (eBioscience, San Diego, CA). Stained samples were analyzed on a 18 color LSR-II Flow Cytometer (BD Biosciences, San Jose, CA); data analysis using FlowJo version 8.7.3 software (FlowJo, Ashland, OR). See Supplemental Methods in S1 File for analytical panels. Tregs were sorted using the following markers PI^-^CD3^+^CD4^+^CD25^+^CD127^-^ on an 18 color FACS Aria-II cell sorter and FACS Diva software (version 7.0; BD Biosciences, San Jose, CA; Fig D, part A in S1 File). The sorted Tregs were at least 95% pure and (98% FoxP3^+^ when stained for FoxP3; Fig D in S1 File). *FOXP3* mRNA levels were consistently high in all samples further confirming that the flow sorted cells were purified Tregs (Fig E in S1 File).

### RNA Isolation

Total RNA was extracted from sorted Treg cells using AllPrep DNA/RNA Micro Kit (Qiagen, Valencia CA) following manufacture’s protocol. RNA quality was assessed by a 2100 BioAnalyzer (Agilent Technologies, Santa Clara CA) at the University of Rochester Genomic Research Center. Total RNA from each sample was used for cDNA synthesis using the Ovation® RNA-Seq, with normalized RNA input for each sample (NuGEN, San Carlos CA). Amplified cDNA product was purified through a MinElute reaction cleanup column (Qiagen). Amplified cDNA quality was determined with a 2100 BioAnalyzer (Agilent Technologies).

### Quantitative Real-Time Polymerase Chain Reaction

Taqman assays for quantification of mRNA for BCL6, CCL20, CCL3, CCR6, CCR7, CXCR4, FOXP3, IL-10 KLF2, S1PR1, SELL and ACTB (beta-actin) were obtained from the Assays on Demand Panel (Life Technologies, Carlsbad CA). All reactions were performed in triplicate with 40 cycles and analyzed on ABI 7900HT real-time PCR instruments (Applied Biosystems, Carlsbad CA) by the Genomics Research Center using ΔΔCt relative quantification method.

### RNAseq Data

Raw sequences were generated using a SOLiD 4 System (Applied Biosystems). 50bp reads were output and demultiplexed according to barcode using SOLiD Instrument Control Software. The resulting sequence reads were aligned to the reference genome (hg19) using Bioscope Software (Applied Biosystems) with default settings. Data were expressed as reads per million mapped reads (RPM). RNAseq data described in this publication have been deposited in NCBI’s Gene Expression Omnibus (GEO) and are available through GEO Series accession number GSE74102 (http://www.ncbi.nlm.nih.gov/geo/query/acc.cgi?acc=GSE74102).

### Cytokine Analysis

Sorted Tregs (Fig D, part B in S1 File) were cultured at 37°C/5% CO_2_ in serum free media stimulated with soluble anti-CD3 (OKT3) and anti-CD28 (CD28.2) monoclonal Abs (1 μg/ml; eBioscience) for 6 hours. Culture supernatants were analyzed at the Roswell Park Cancer Institute Flow & Image Cytometry Facility (Buffalo NY) for IL-10, MIP-1α (CCL3), MIP-1β (CCL4) (MILLIPLEX MAP Human High Sensitivity T cell Panel, EMD Millipore, Billerica MA), BCA-1 (CXCL13) and IL-16 (MILLIPLEX MAP Human Cytokine/Chemokine Magnetic Bead Panel II, EMD Millipore) on a Luminex 100 analyzer (Luminex, Austin TX). Analysis performed in duplicate.

### Cell Migration Assay

SCSs were depleted of dead cells using the Dead Cell Removal Kit (Miltenyi Biotech, Bergisch Gladbach Germany) followed by human CD4 T cell isolation by negative magnetic selection using the Human CD4+ T cell Isolation (Miltenyi Biotech). CD4 T cells were cultured at 37°C/5% CO_2_ in RPMI 1640 medium containing 10% FBS overnight. Transwell chemotaxis assays were performed using 24-well plates with 5-μm pore size inserts (Corning, Tewksbury MA). Cells were equilibrated at 37°C/5% CO_2_ in migration medium (RPMI 1640, 1% BSA, 10 mM HEPES, 1% pen-strep/L-glutamine) at 1 × 10^6^ cells/ml for 1h before use. A total of 500 μl chemoattractant in migration medium was applied to the lower chamber and 200 μl cells applied to the upper chamber. Chemokines used were 1000 ng/ml CXCL13 and 25ng/ml SDF-1α (PeproTech, Rocky Hill NJ) and 1nM D-erythro-S1P (Avanti Polar Lipids, Alabaster AL). After 2 h at 37°C/5% CO_2_, inserts were discarded and cells from the lower chamber including input samples were stained with CD3, CD4, CD25 and CD127 Abs. Each sample had 50 μl Accucount beads (5.1-μm diameter; Spherotech, Lake Forest IL) added before analysis for quantitation by flow cytometry. Percent migration was determined as 100 × ([Tregs in lower chamber/bead events in lower chamber]/[input Treg events/input bead events]) and then the % baseline migration to media only was subtracted. Duplicate samples were run.

### Statistics and Expression Analysis

Significant differences for flow, Luminex, and qPCR data were assessed using Wilcoxon rank sum tests with permutations to compute p-values. Data from the migration assay were analyzed using paired *t*-tests after applying a log transformation to normalize data. Only genes that had an RPM value > 3 for at least one sample were retained in subsequent analyses (n = 14725). Resulting expression measurements were log-transformed after adding “1” to values. Associated group comparison p-values were computed using 10,000 permutations. Genes were selected using false discovery rate (FDR) with a threshold of 0.05 for NLN vs. FL and RLN vs. FL, and 0.10 for RLN vs. NLN. Clustering, heat map generation, principal components and other analyses were performed using scripts written in Matlab (The Mathworks Inc., Natick MA, www.mathworks.com). Functional enrichment, pathway and upstream regulator analyses were performed with Ingenuity Pathway Analysis (IPA, Qiagen, Redwood City CA, www.ingenuity.com).

## Results

### Gene Expression Differences between FL, RLN, and NLN Tregs

**Fig 1:**
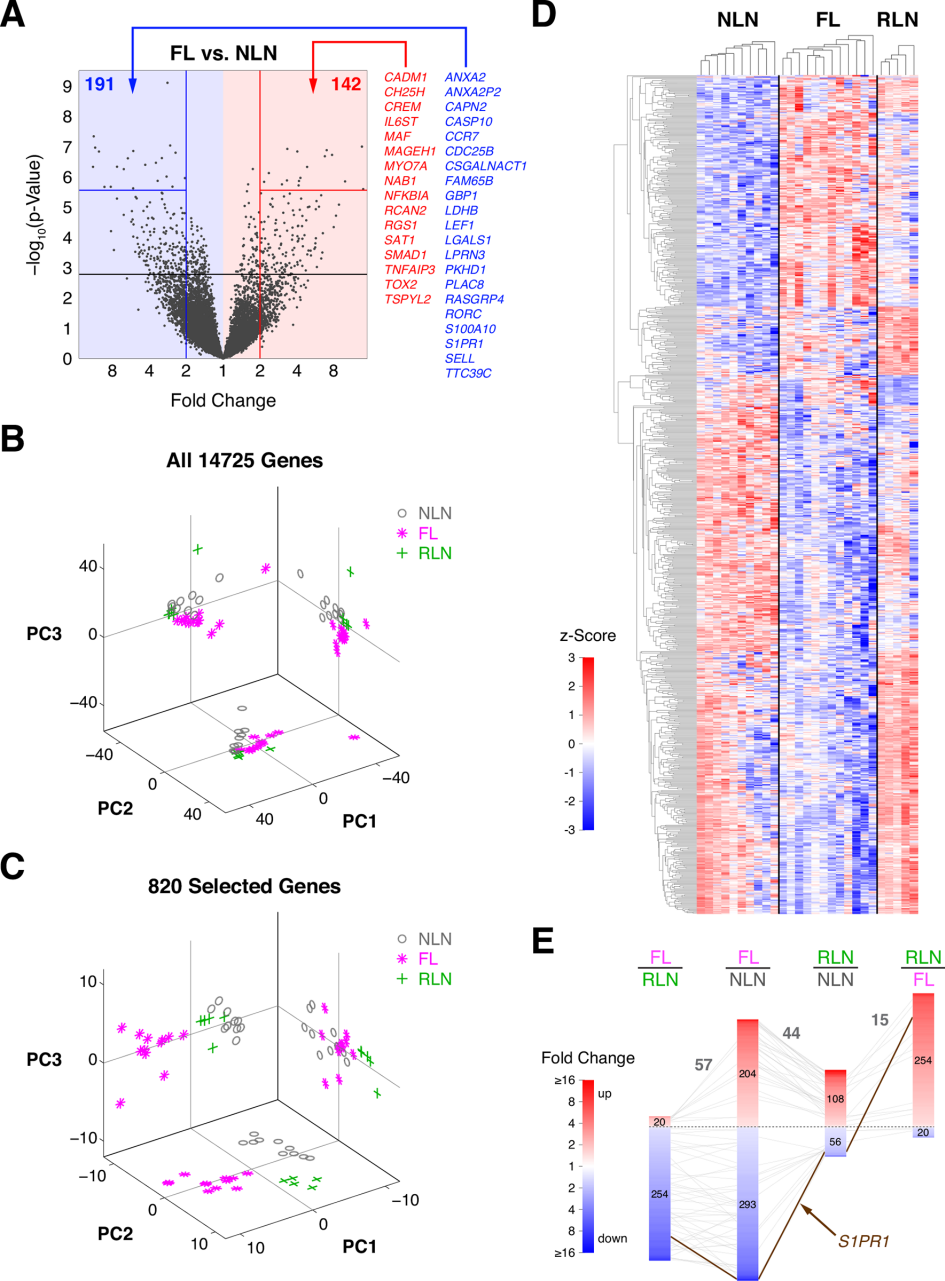
Significant genes between groups and feature selection. A. Volcano plot for FL vs. NLN comparison. -log_10_ p-value is plotted against the fold change (log_2_ scale). Black horizontal line corresponds to an FDR of 0.05. 37 genes with a FDR < 0.001 (horizontal red and blue lines) and a greater than two-fold change in either direction are labeled. Red and blue numbers indicate number of genes with FDR < 0.05 and a two-fold change. See Supplemental Fig 2 for similar plots of RLN vs. NLN and RLN vs. FL. B. Distribution of 27 samples over the first three principal components (PCs). PCs were computed using log_10_-transformed RPM to reduce the dimensionality. PCs were based on all 14725 genes. Shadows of three-dimensional data are shown on the three coordinate planes. C. PCs computed considering only 820 selected genes. These genes were significant for at least one pairwise comparison (FL vs. NLN, n = 497; RLN vs. FL, n = 274; RLN vs. NLN, n = 164). D. Heat map of clustered, significant genes as described in C. Rows are genes, columns are samples, and color maps to a gene’s z-score based on log-transformed data (computed independently for each gene). Samples are organized by histology but are clustered within their group. Euclidean distance and average linkage were used for clustering. E. Comparison of three statistical gene lists. Each bar represents a comparison with their constituent genes ranked according to fold change (color) and with the number of up- and down-regulated genes shown in the red and blue portions, respectively. Lines connecting bars represent genes shared between lists. (RL vs. FL is shown twice but with different polarities). Only one gene, S1PR1 appeared in all three lists (highlighted in brown).

We found 820 genes out of 14725 that were significantly differentially expressed in at least one comparison (FL vs. NLN, RLN vs FL, and RLN vs. NLN). Fig 1A illustrates the relationship between unadjusted p-value and fold change for all genes in the FL vs. NLN comparison (Fig A in S1 File shows all comparisons). We found 497 genes differentially expressed in FL vs. NLN, with most of them being down-regulated (293 lower in FL, 204 higher; 191 and 142, respectively, having greater than 2-fold change) with FDR < 0.05. In contrast, a majority of differentially expressed genes were up-regulated in RLN vs. NLN (164 total: 108 higher in RLN, 56 lower with FDR < 0.10; no genes selected at FDR < 0.05). Most differentially expressed genes were higher in RLN relative to FL at FDR < 0.05 (274 total: 254 higher in RLN, 20 lower).

Fig 1B shows the first three principal components (PC) for all 27 samples, as projections of the 3-D data onto the three bivariate planes to visualize group differences. Fig 1C shows a similar plot only considering 820 selected genes described above. Both plots indicate separation between groups, even when PCs were computed with all genes. In that case, separation of the sample groups was observed between NLN and FL, notably along PC3 (Fig 1B) indicating distinct transcriptional profile-wide differences. Fig 1D shows a heat map for the 820 genes where samples are clustered within each group, suggesting that the largest separation was found between FL and NLN samples. RLN samples differed to a lesser extent from both FL and NLN samples, while also exhibiting commonalities in gene expression with both of these two groups.

Fig 1E shows overlap in significant gene lists along with relationships in directions of changes of genes. 44 genes that were significant in both the FL vs. NLN and RLN vs. NLN comparisons, changed in the same directions (relative to NLN). Remarkably, the only gene common to all three lists was S1PR1 where expression in NLN > RLN > FL (Fig 1E).

### Alterations in Lymphocyte Trafficking and Nodal Retention

Table A in S1 File shows the top 25 significant up- and top 25 significant down-regulated genes based on FDR. In FL vs. NLN, 6 of the 25 down-regulated genes are known to be involved in lymphocyte migration and trafficking. No such genes were identified in either of the other two comparisons. Noteworthy genes include S1PR1, the receptor for sphingosine-1-phosphate (S1P), an extracellular lipid mediator critical for lymph node T cell egress, L-selectin (SELL), a lymphocyte homing receptor that facilitates T-cell migration from the periphery into secondary lymph node structures, and chemokine receptor 7 (CCR7) which controls the migration of T cells into inflamed tissues and the nodal T cell zone.

### Functional Annotation Differences Correlating with Migration and Cell Cycle

**Fig 2:**
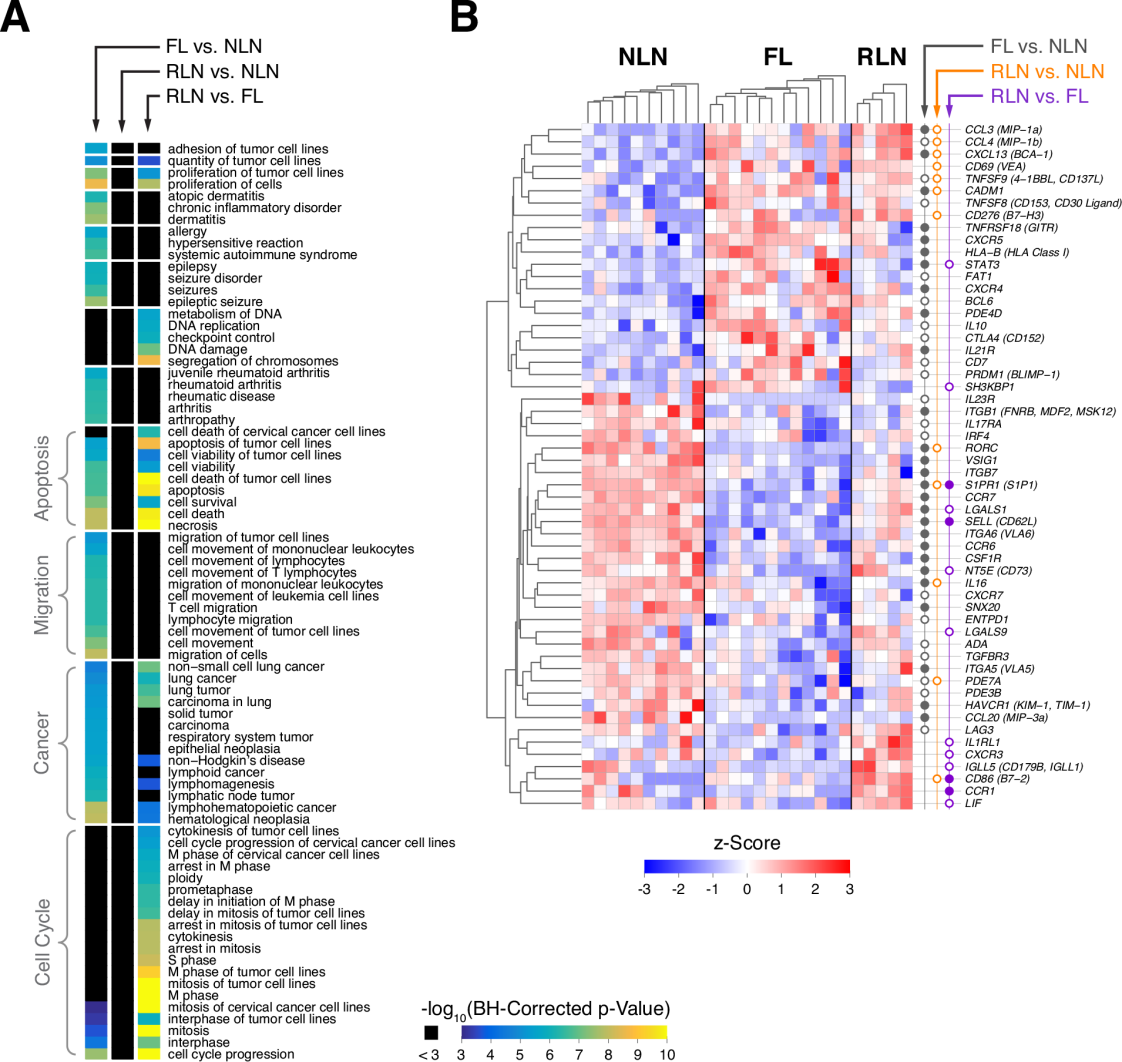
Characteristics of significant gene lists and functionally relevant genes. A. IPA enriched functions for significant genes for the three pairwise comparisons. Functions that had a B-H corrected enrichment p-value less than 0.00001 for at least one comparison are shown. Functions were clustered to aid in visualization based on their patterns of category membership (a function can be associated with or more broader category) using one minus the kappa statistic for distance (dendrogram not shown). In the heat map, functions within each cluster (delineated by white lines) are sorted by size, their significance in the FL vs. NLN gene list, and then the significance in the RLN vs. FL gene list. No enriched functions were found at this significance level for the RLN vs. NLN list at this level of significance (middle column). B. Heat map of genes in categories from Table 1 that had a pairwise difference with FDR < 0.1 for at least one comparison. Color maps to a gene’s z-score based on log-transformed data (computed independently for each gene). Genes and samples (within groups) are clustered using Euclidean distance and average linkage. Circles next to the heat map indicate whether the gene had a FDR < 0.1 (unfilled) or < 0.05 (filled) for the specified comparison.

Fig 2A shows enriched functional annotations (from significant gene lists). Rows represent annotations enriched at a Benjamini-Hochberg corrected p-value of less than 0.00001 for at least one comparison. Notably, FL vs. NLN genes were enriched for cancer and migration categories while there was almost no representation of those for RLN vs. FL. Similarly, RLN vs. FL genes showed enrichment for cell cycle categories while there was almost no enrichment in the FL vs. NLN list. No enriched functions were found for RLN vs. NLN. This marked difference in enriched functional annotation is consistent with relatively small overlap between these two gene lists (Fig 1E). Results for IPA canonical pathways and upstream regulators analyses are shown in Table B in S1 File and Fig B in S1 File.

### Alterations in Lymphocyte Trafficking, Nodal Retention and Suppressive Capacity

We compiled lists of genes in 14 migration related categories (Table 1) motivated by enrichment of "cell migration" annotations, and by the significant down-regulation of S1PR1 and L-selectin, both in FL vs. NLN Tregs. As we have previously shown that FL Tregs are more potent suppressors than those derived from NLN,[10] we included genes that encoded for proteins mediating Treg suppression. Gene expression differences of the categories’ member genes as shown in Fig 2B. Most of these genes were significant for FL vs. NLN at FDR < 0.1 or 0.05, whereas a minority were significant for the other comparisons.

**Table 1:** Gene list categories.

Gene List Name	Total	RNAseq	FL vs NLN	RLN vs NLN	RLN vs FL
B7/CD28 Family	15	13	0	2	1
Cadherins	118	53	0	0	0
Constitutively Expressed Treg Antigens	3	3	1	0	0
Cytokines, Chemokines and their Receptors	228	152	11	5	1
Ig Superfamily Cell Adhesion Molecules	66	45	2	1	0
Integrins	27	23	4	0	0
Mediators of Treg Suppression	59	39	4	2	1
Other Co-Stimulatory Molecules	106	94	6	4	2
Reg. of T Cell Co-Stimulation by TNF Superfamily Members	23	18	1	1	0
SLAM Family	11	10	0	0	0
Selectins	11	10	2	0	1
Syndecans	6	4	0	0	0
Treg Homing and Retention	8	8	6	1	2
Treg Subsets	6	6	2	1	0

For each of 14 categories, the number of: total number of genes, number of genes expressed in our RNAseq data, and genes that are significant at FDR < 0.05 (for FL vs. NLN and RLN vs. FL) or FDR < 0.1 (for RLN vs. NLN).

**Fig 3:**
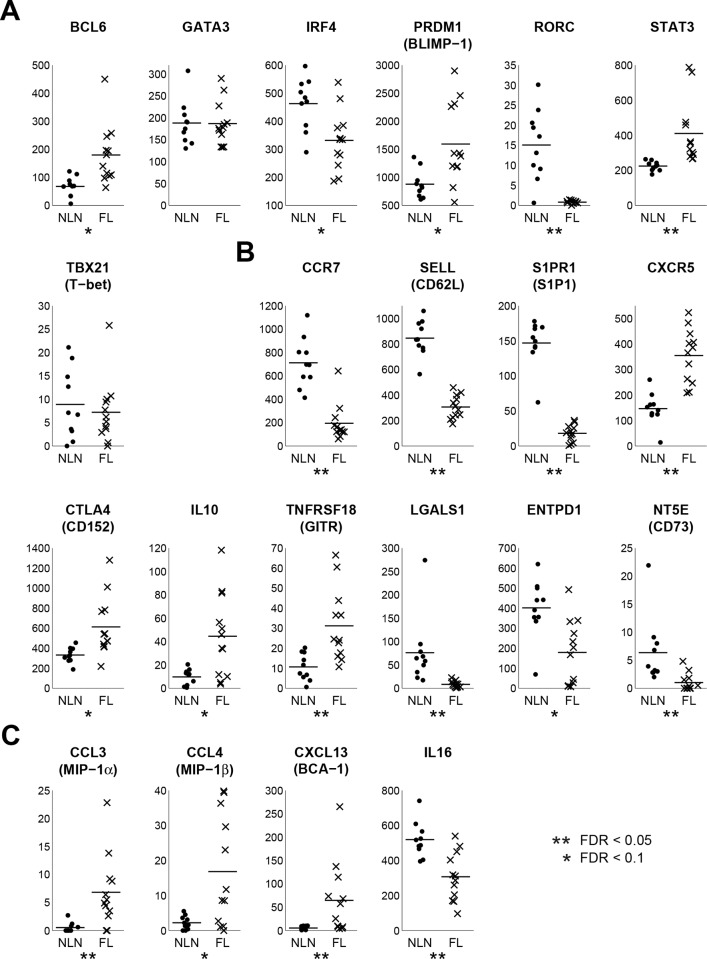
Gene expression scatter plots of selected genes by category. RPM data are not log-transformed for these plots. Line segments are group means. A. Treg subset genes. B. Treg homing and retention genes. C. Immune cell recruitment genes.

Expression of 21 differentially expressed genes related to Treg subsets, homing, retention and immune cell recruitment as well as suppressive capacity is shown in Fig 3. FL has more Tregs that express genes consistent with T-follicular regulatory cells (TFR), CD3^+^CD4^+^CXCR5^+^PD1^+^CD25^+^BCL6^+^FoxP3^+^, a population recently characterized in the FL microenvironment that suppress the function of TFH.[8, 22–26] Whereas NLN Tregs had variable expression of RORC, there was essentially no RORC expression in FL Tregs. As RORC-expressing Tregs produce IL-17,[33] this suggests that in contrast to NLN, there are no IL-17 producing Tregs in the FL microenvironment. Furthermore, our RNAseq results also support published data[10, 34–41] suggesting that FL Tregs are more suppressive than NLN Tregs with increased expression of CTLA4, IL-10, and GITR. Expression of both CD39 (ENTDP1) and CD73 were reduced in FL derived Tregs as compared to NLN.

CCR7 was down-regulated in FL Tregs relative to NLN suggesting that FL Tregs are less likely to reside in the T cell zone of the lymph node. CXCR5 is up-regulated at the transcriptional level (consistent with TFR phenotype) compared to NLN Tregs which facilitates migration of these cells to the GC in response to a CXCL13 gradient within the GC. Additionally, FL Tregs express CXCL13 suggesting the existence of an autocrine feedback resulting in Treg retention in the GC. Finally, there is a striking decrease of S1PR1 in FL Tregs compared to NLN Tregs. These results suggest, along with the nodal egress of T cells from S1PR1 binding to its cognate ligand S1P, the transcription profile of FL Tregs favors the localization and retention of Tregs into the GC of the malignant lymph node. Finally, FL Treg retention is also supported by the finding that L-selectin (SELL) expression in FL Tregs is lower than that of NLN Tregs.

To validate RNAseq data we performed qPCR analysis of 11 of the 820 significant genes (Table C in S1 File). Five of these that were found differentially expressed in FL vs. NLN based on the RNAseq data remained significantly differentially expressed by qPCR (p ≤ 0.033), and followed the same trend; those that did not reach statistical significance changed in the same direction as in the RNAseq data.

### Protein Expression Highlights Alterations in Lymphocyte Trafficking, Nodal Retention and Suppressive Capacity

**Fig 4:**
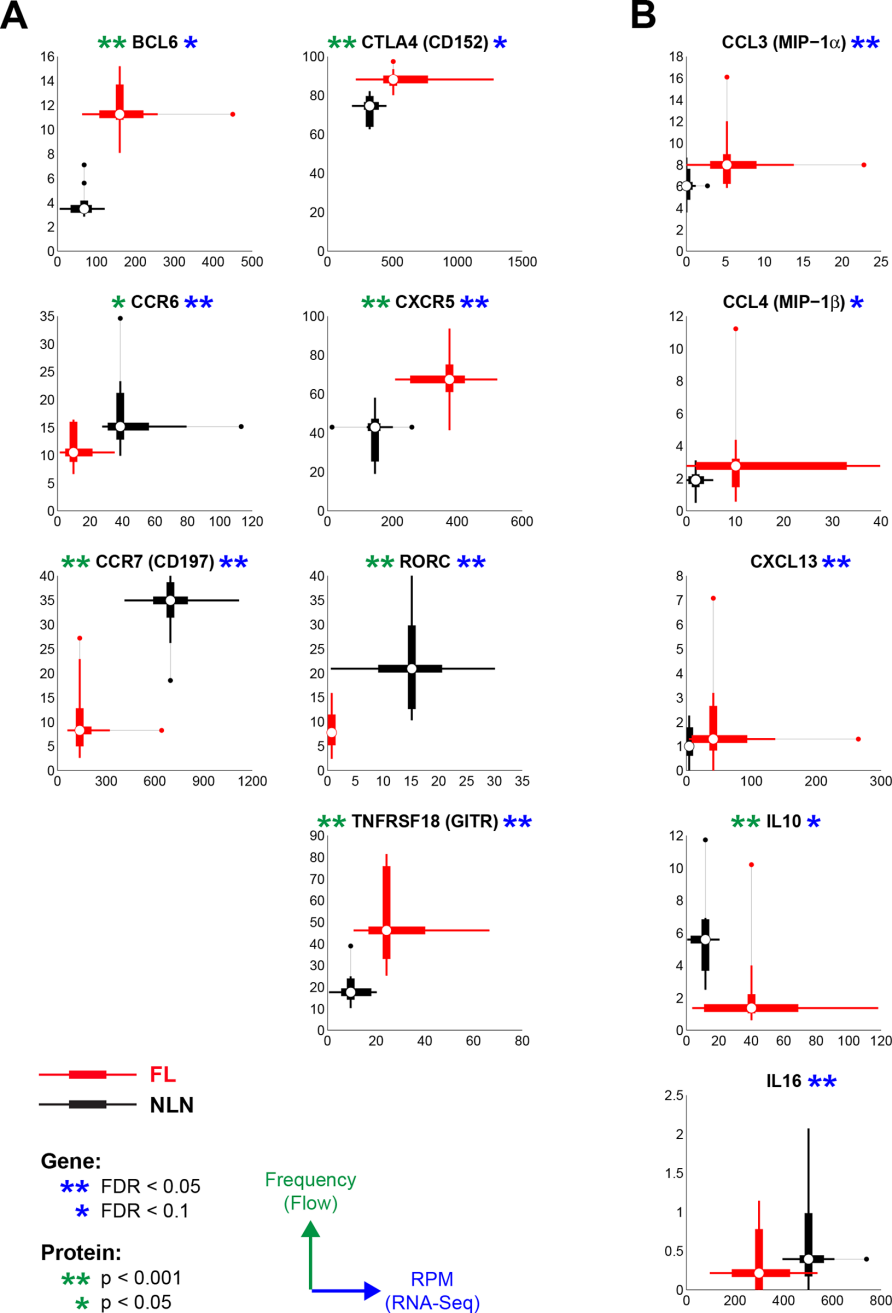
Comparison of gene and protein expression for selected genes. Two-dimensional box-plots are shown for NLN (black) and FL (red) for gene expression data (not log-transformed, x-axis) and flow cytometry data (frequencies, y-axis). White circles represent the median values, thick lines represent the range between the 25^th^ and 75^th^ percentiles, and thinner lines show the extent of the non-outlier data. Outliers are dots and are defined as points at least 1.5 times the interquartile range beyond either the 25^th^ or 75^th^ percentiles. For gene expression data, n = 10 (NLN) and n = 12 (FL). For flow cytometry data, n = 10 for both NLN and FL. Gene expression data and flow cytometry data are from different sample sets, except for four NLN samples in common. Significant differences in gene expression are based on FDR (see Methods).

**Fig 5:**
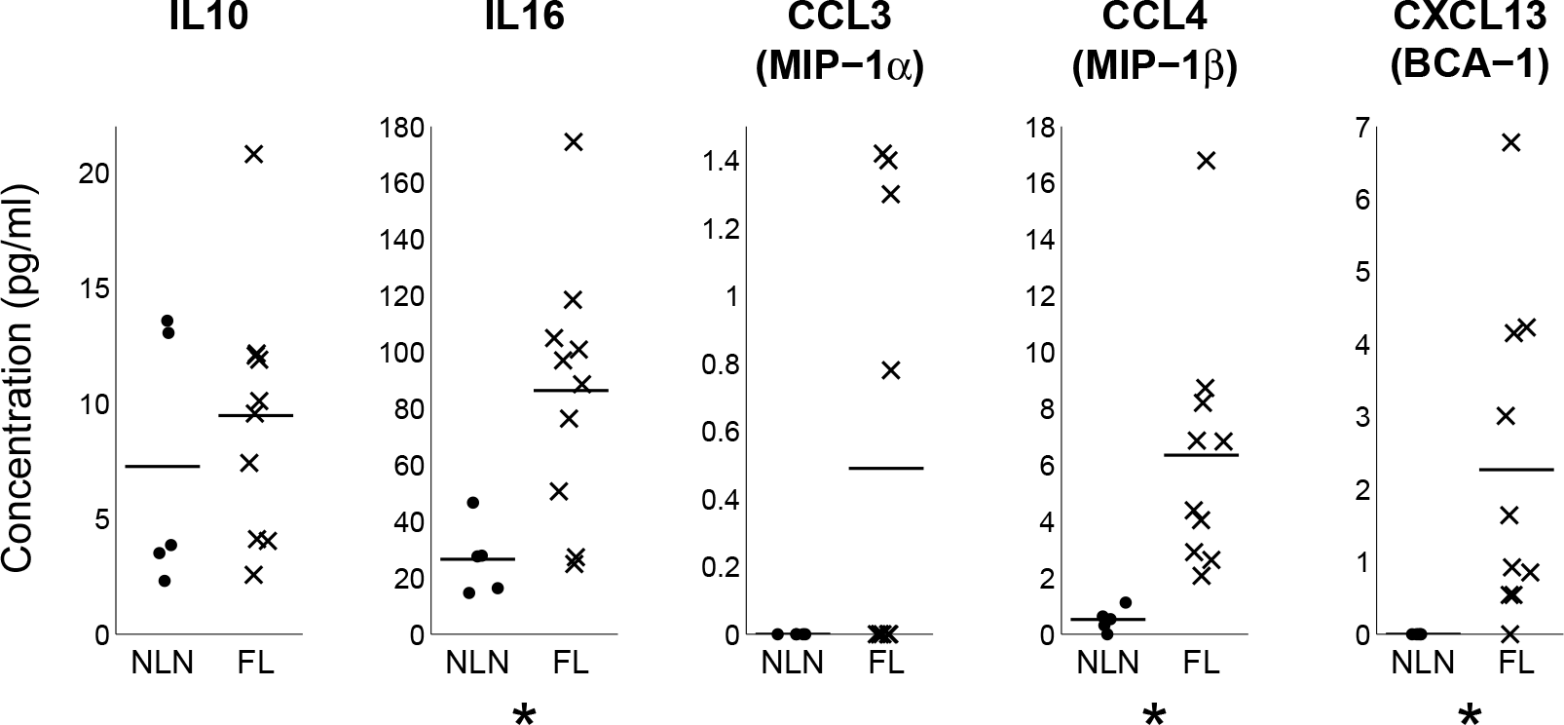
FL Tregs secrete significantly higher levels of selected cytokines and chemokines than NLN Tregs. Tregs were sorted from FL (n = 10) and NLN (n = 5) samples and were cultured for six hours in serum free media with added antiCD3/CD28. Culture supernatants were collected and analysed for IL-10, IL-16, CCL3, CCL4, and CXCL13 by luminex. Assays were performed in duplicates. Line segments represent mean values for each group. Values below the limit of detection are set to “0”. Significance indicated by asterisks (p < 0.05) determined by Wilcoxon rank sum tests with permutations to compute p-values.

Fig 4 shows protein expression of several genes by flow cytometry in FL and NLN Treg combined with corresponding gene expression data, some of which is shown in Fig 3. All cell surface markers tested (CCR6, CCR7, CTLA4, CXCR5 and TNFRSF18) as well as the transcription factors (BCL6 and RORC) were statistically significant in both RNAseq and flow data (Fig 4A). However most of the intracellular cytokines, except for IL-10, were not significantly different between FL and NLN Tregs at the protein level (Fig 4B). Furthermore, based on multiplex immuno-assays, CCL4, IL-16 and CXCL13 are secreted at significantly higher levels in FL vs. NLN Tregs (Fig 5). CCL3 and IL-10 follow the same trend, though not significant, which may be partially due to low number of NLN Tregs. Together, these results validate the RNAseq data at the protein level.

**Fig 6:**
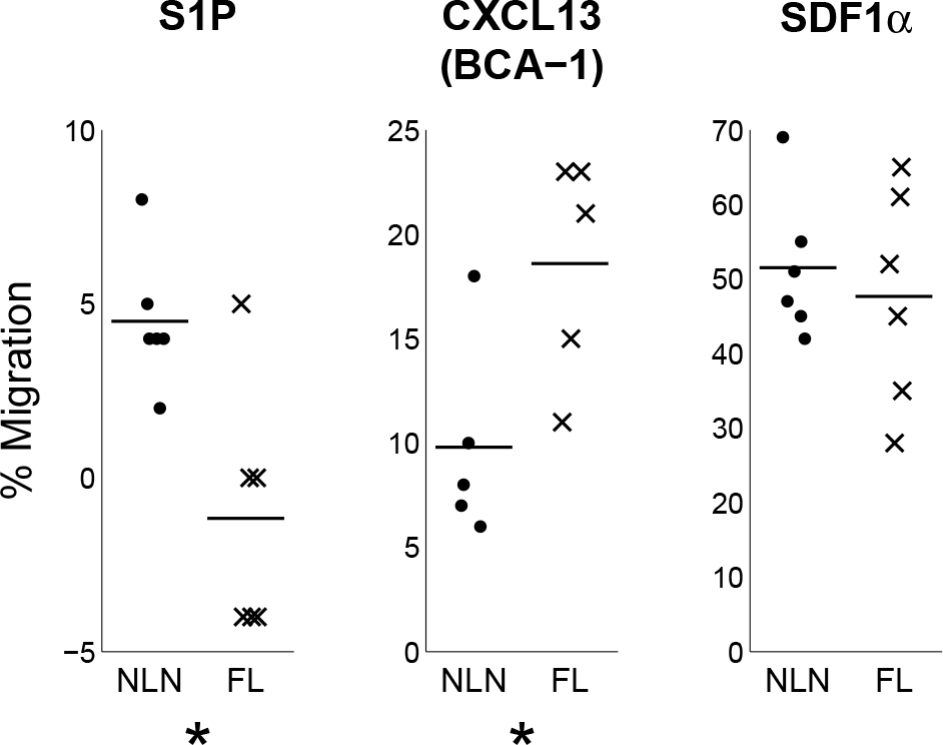
FL Tregs, in contrast to NLN Tregs, readily migrate to CXCL13 but not to S1P. CD4 T cells were isolated by negative selection from FL (n = 6) and NLN (n = 6) samples and were rested overnight. Transwell chemotaxis was performed as described in the experimental procedures section using 1000 ng/ml CXCL13, 25 ng/ml SDF-1α and 1nM D-erythro-S1P. Cells that migrated were surface stained for CD3, CD4, CD25 and CD127 and were analyzed by flow cytometry using Accucount beads for quantitation. Assays were done in duplicates for each sample. Line segments represent mean values for each group. Significance indicated by asterisks (p < 0.05) determined by paired t-test following log-transformation of variables (5 was added to S1P to obtain positive values).

Fig 6 provides functional evidence that transcriptional differences of FL Tregs are related to their migration to and retention in the lymph node follicle by showing that FL Tregs migrate significantly more than NLN Tregs to CXCL13 (CXCR5 ligand) and that they were completely unresponsive to S1P (S1PR1 ligand), the latter suggesting that their egress from the lymph node will also be significantly impaired. As a positive control, migration to SDF1α is unchanged in FL relative to NLN Tregs, demonstrating that these Tregs do not exhibit global alteration in their ability to migrate to a common chemoattractant.

To understand whether down-regulation of S1PR1 in FL Tregs is reversible when separated from their microenvironment, Tregs from four different FL samples were sorted and cultured for up to 48h in complete media. Using RT-PCR, expression of S1PR1 did not change even at 48h (Fig C in S1 File).

## Discussion

**Fig 7:**
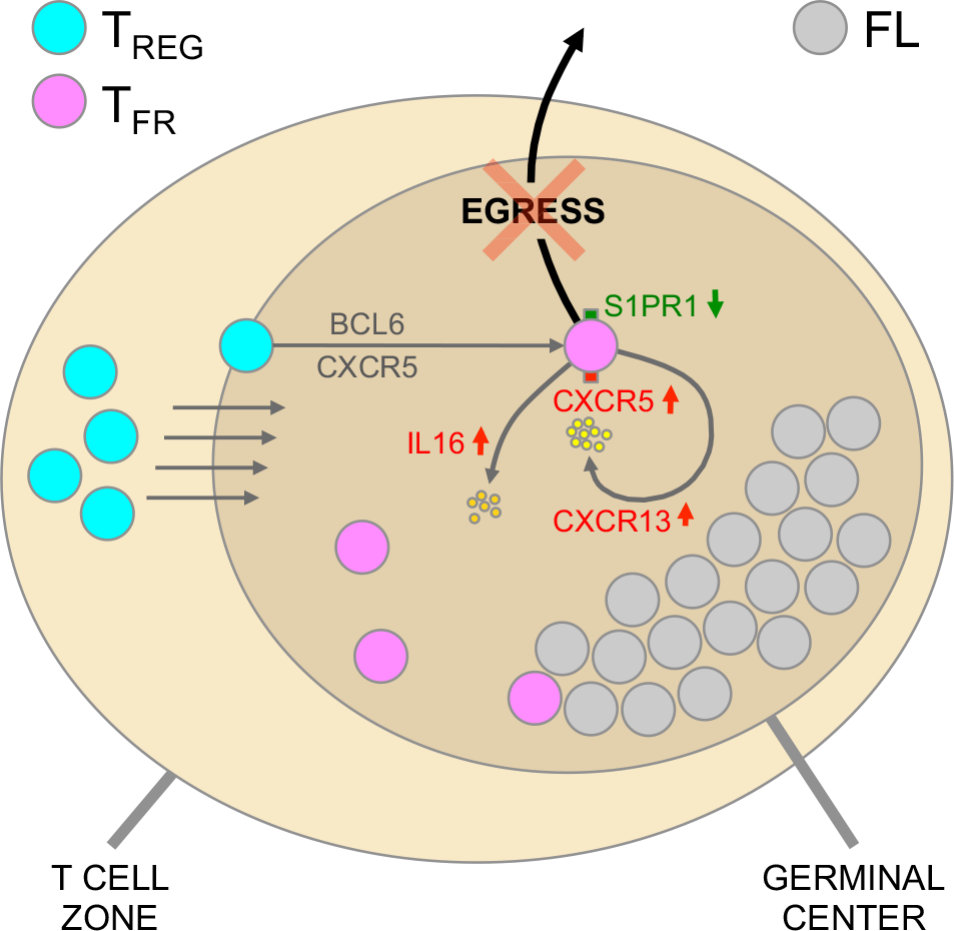
FL Treg Infiltration, Localization and Retention. Schematic representation of the various molecules identified that play a role in the accumulation, localization and nodal retention of FL Tregs. Increased expression of CXCR5 and CXCL13 would be likely to result in B cell follicle localization and accumulation of FL Tregs. Also, CXCR5-CXCL13 would function as an autokrine loop to futher retain FL Tregs within the follicle. Increased secretion of IL-16 by these Tregs would result in further recruitment of additional Tregs. Lastly, nodal egress of FL Tregs would be inhibited by the decreased expression of S1PR1, additionally resulting in nodal accumulation of FL Tregs.

Although expression level of FOXP3 does not differ significantly between Tregs infiltrating FL, NLN and RLN, differences in the global transcriptome profile exist between these populations, mostly between FL and NLN. Further, we found that cell migration and movement categories were highly enriched based on the FL vs. NLN comparison. Consistent with this, significant differences were found in chemokine, chemokine and G-protein receptors, and adhesion molecule genes in FL vs. NLN Tregs, including CXCL13, CCR7, CXCR5, S1P1 and L-selectin. These differences suggest a propensity for GC localization and nodal retention of FL Tregs (Fig 7). Our data support the novel finding that FL Tregs have the ability to “auto-regulate” their own chemotaxis via a CXCL13-CXCR5 autocrine loop.

Previously, we found a greater proportion of T-cells having a Treg phenotype in FL compared to NLN partially due to FL tumor cell elaboration of CCL22 which is chemotactic for CCR4 expressing Treg-cells.[14] Present data suggest an additional novel mechanism, namely, the lack of expression of S1P1 in FL but not NLN Tregs, a type 1 G-protein coupled receptor that is a primary determinant for lymphocyte egress from the lymph node. S1P1 mediates lymphocyte egress from the thymus and secondary lymphoid organs likely through internalization of the receptor upon agonist stimulation.[42] Because there is no optimal antibody to characterize human S1P1 expression at the protein level, we confirmed our findings functionally by demonstrating that Tregs from six different human FL samples were not able to migrate in response to S1P, unlike in NLN. Although recently shown to mediate Treg accumulation in a murine breast cancer model,[43] this is the first demonstration to our knowledge that S1P1 loss may be a mechanism for Treg accumulation in human tumor.

Microenvironmental signals may transiently alter expression patterns and functional capacity of infiltrating lymphocytes, and removal from this environment may support reversion to normal patterns. Local signals may account for the decrease in S1P1 expression, thus temporally retaining these cells within the tumor microenvironment. However, the decrease in S1P1 expression does not appear transiently inhibited by the microenvironment, as removing FL Tregs from the microenvironment and culturing them for up to 48 hours *in vitro* does not result in increased S1P1 expression. This suggests a more permanent expression pattern conferred on tumor infiltrating Tregs and consequently, a more terminal differentiation pattern. Further study into mechanisms by which FL Tregs acquire these unique expression patterns that facilitate increased tumor localization as well as retention within the malignant node would undoubtedly result in additional therapeutic targets.

Our studies further show that FL Tregs express a cytokine/chemokine profile that is unique compared to NLN Tregs, and likely plays a fundamental role in shaping the FL tumor microenvironment. For example, we show that IL-16 is abundantly secreted by FL Tregs compared to NLN (Fig 5). IL-16 has chemo-attractant activity with preferential recruitment of the Th1 and Treg subsets.[44] Although FL Tregs produce more IL-16 then do NLN Tregs, NLN Tregs have more IL-16 transcripts. This discrepancy is likely due to mature IL-16 requiring processing of a precursor molecule.[45, 46] Finally, we demonstrate that FL Tregs produce CCL-3 and CCL-4, both of which are chemotactic for CCR5 expressing Tregs and CD4 and CD8 effector cells, more so than NLN Tregs. Taken together, the different transcriptional profile of FL vs. NLN Tregs leads to different cytokine and chemokine profiles that have both an autocrine effect on Tregs as well as possible effects on different immune effector cells.

Not all gene expression data we observe directly correlates with protein levels, as described for IL-16 above. We have previously shown with flow cytometry that there was no statistically significant difference in the levels of either CD39 or CD73 protein, on a per cell basis,[11] however, our data here demonstrates reduction in CD39 (ENTDP1) and CD73 transcripts in FL Tregs as compared to NLN. This discrepancy has at least two potential explanations: First, intracellular pools of these proteins may differ between different Tregs while surface expression remains similar (we only assessed extracellular protein levels by flow). Second, global mRNA and protein levels do not necessarily correlate.[47–49]

We have previously shown that FL Tregs show greater suppressive capacity then those derived from NLN or RLN.[10] As shown, FL Tregs express higher levels of IL-10, CTLA-4 and GITR then do NLN Tregs at both transcriptional and protein levels, consistent with this increased suppressive capacity. This is of particular interest as therapeutic agents targeting each of these inhibitory pathways are at various stages of development. Additionally, it has recently been shown that signaling through Treg S1P1 results in an AKT mediated attenuation of Treg suppression.[50] Because FL Tregs have lower expression of S1P1 then do NLN Tregs, they may be subjected to less attenuation of their suppressive function resulting in greater effector T-cell suppression then seen with NLN Tregs.

There is great interest in targeting Tregs therapeutically to augment immunotherapeutic approaches such as tumor vaccines or checkpoint inhibitors. Because our data suggest that FL Tregs are skewed towards a TFR phenotype, along with recent findings that suggest Tfh support FL cell viability (through CD40L expression and elaboration of IL-4),[51] it is possible that depletion of Tregs in FL could have an adverse clinical effect by removing the “brakes” on the FL supporting TFH. Furthermore, given cytokines/chemokines elaborated by FL Tregs, it is likely that Treg depletion would have additional effects on remodeling tumor microenvironment, which may or may not be clinically beneficial. Understanding how microenvironment of a given tumor type modulates the function of Tregs infiltrating the tumor will be critical to determining whether Treg depletion is a rational therapeutic strategy for a given tumor.

Our data support shaping of the tumor infiltrating Treg transcriptome by the tumor microenvironment. Further insights into pathways through which tumors modulate the Treg transcriptome and thus the function of tumor infiltrating Tregs could lead to discovery of new therapeutic targets.

## Supporting Information

S1 File Supplemental Methods, Figures and Tables. (PDF)

S2 File Supporting Data. File containing data for flow cytometry, Luminex, migration and S1P1 qPCR assays.
(PDF)
