# Supplementary material for: Follicular Lymphoma Tregs Have a Distinct Transcription Profile Impacting Their Migration and Retention in the Malignant Lymph Node
Source: PLoS One. 2016 May 26;11(5):e0155347. doi: 10.1371/journal.pone.0155347 (PMC4882026; doi:10.1371/journal.pone.0155347)
Supplement: S1 File — (PDF) [file pone.0155347.s001.pdf]

Supplemental Materials for Nedelkovska et al.

SUPPLEMENTAL METHODS

Details for Flow Cytometry Panel

The following analytical panels were used for verification of several markers identified by the RNAseq analysis. Regulatory T cell phenotype panel: surface antibodies, GITR PerCP-eFluor710 (eBioscience, San Diego, CA), CD25 BV421, CD3 BV570 (Biolegend, San Diego, CA), CD152 APC, CD16 PE-Cy5, CD197 PeCy7 (BD Biosciences, San Jose CA), $S1P_1$ PE (R&D Systems, Minneapolis, MN), CD4 PE-TR, CD14 Tricolor (Invitrogen, Carlsbad, CA). Intracellualr antibody, FoxP3 Alexa488 (eBioscience, San Diego, CA). T cell chemokine receptor/transcription factor panel: surface antibodies, CD25 APC-Cy7, CXCR3 AF700, CXCR5 AF657, CCR6 PerCP-Cu5.5, PD-1 BV421 (BD Biosciences, San Jose, CA), CD4 BV605, CD3 BV570 (Biolegend, San Diego, CA), CCR4 FITC (R&D Systems, Minneapolis, MN). Intracellular antibodies, FoxP3 PE-Cy7, RORγt PE (eBioscience, San Diego, CA), Bcl-6 PE-CF594 (BD Biosciences, San Jose, CA). Intracellular cytokine panel: surface antibodies, CD3 BV570 (Biolegend, San Diego, CA), CD4 PE-TR (Invitrogen, Carlsbad, CA), CD25 APC-Cy7 (BD Biosciences, San Jose CA). Intracellular antibodies, FoxP3 Alexa488 (eBioscience, San Diego CA), IL-10 BV421 (Biolegend, San Diego, CA), CXCL13 Alexa700 (R&D Systems, Minneapolis, MN), Mip1α APC, Mip1β PE-Cy7, IL-16 PE (BD Biosciences, San Jose CA). Cell sorting staining, the following fluorochrome-conjugated antibodies were used for cell surface staining: CD3-PE-Cy5, CD4-FITC, CD25-APC, CD25-BV421, CD127-PE (BD Biosciences, San Jose, CA), CD3-APC-Cy7, CD4-BV711, CD127-BV605 (Biolegend, San Diego, CA). Either PI or DAPI were used for viability staining (BD Biosciences, San Jose, CA).

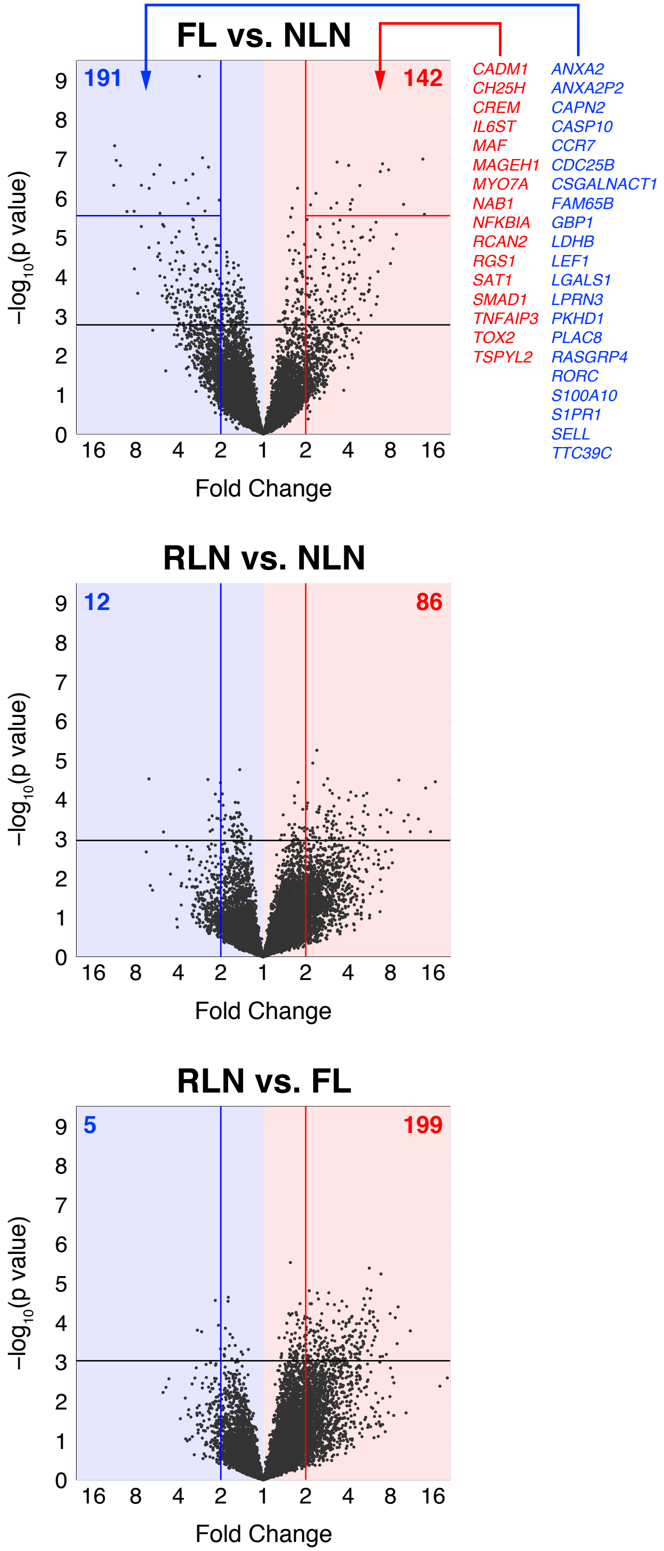

Figure A. Volcano plots for all pairwise comparisons. See legend for Figure 1. Horizontal black lines correspond to FDR < 0.05 for FL vs. NLN and RLN vs. FL, and FDR < 0.1 for RLN vs. NLN (see Methods).

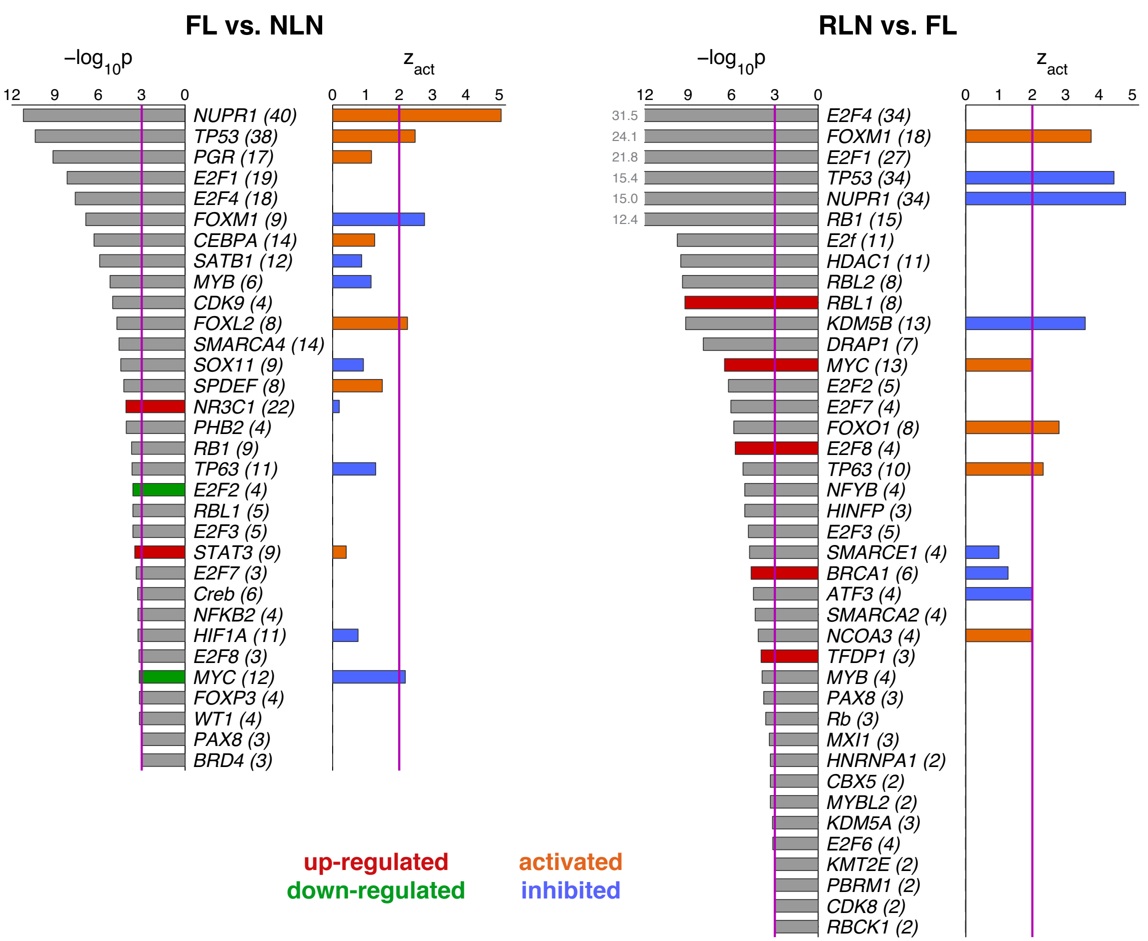

Figure B. IPA upstream analysis. Predicted upstream regulators based on selected gene lists. For the FL vs. NLN colparison (left), 32 upstream regulators were found whose targets significantly overlapped ($p < 0.001$) with the 498 genes supplied at FDR < 0.05. For the RLN vs. NLN comparison (not shown), three regulators were found given a list of 164 genes where FDR < 0.1. For the RLN vs. FL comparison (right), 40 regulators were found given a list of 278 genes where FDR < 0.05. The number in parentheses next to each regulator name is the number of targets in the supplied gene list. For each comparison, overlap p-values are shown as gray bars. If the upstream regulator itself was in the gene list, it is shown shaded as red or green for up- or down-regulated, respectively. Also shown are the activation z-scores that reflect the degree to which the direction of change in the overlapping targets is consi tent with either an activated (orange) or inhibited (blue) state of the regulator. IPA uses a z-score threshold of 2.0 to characterize a regulator as activated or inhibited.

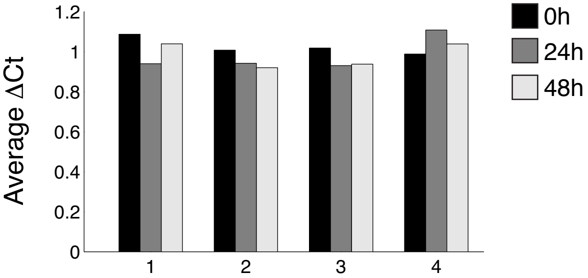

Figure C. FL Tregs are irreversibly modified in the tumor microenvironment. Sorted FL Tregs (n=4) were cultured in complete media for 0, 24, and 48h. Total RNA from each sample was used for cDNA synthesis as described in the experimental methods section. S1PR1 was quantified using qRT-PCR. Shown is the average of three replicates for each sample. There was no significant effect of time ($p = 0.18$) based on a linear mixed model with time as a predictor.

A

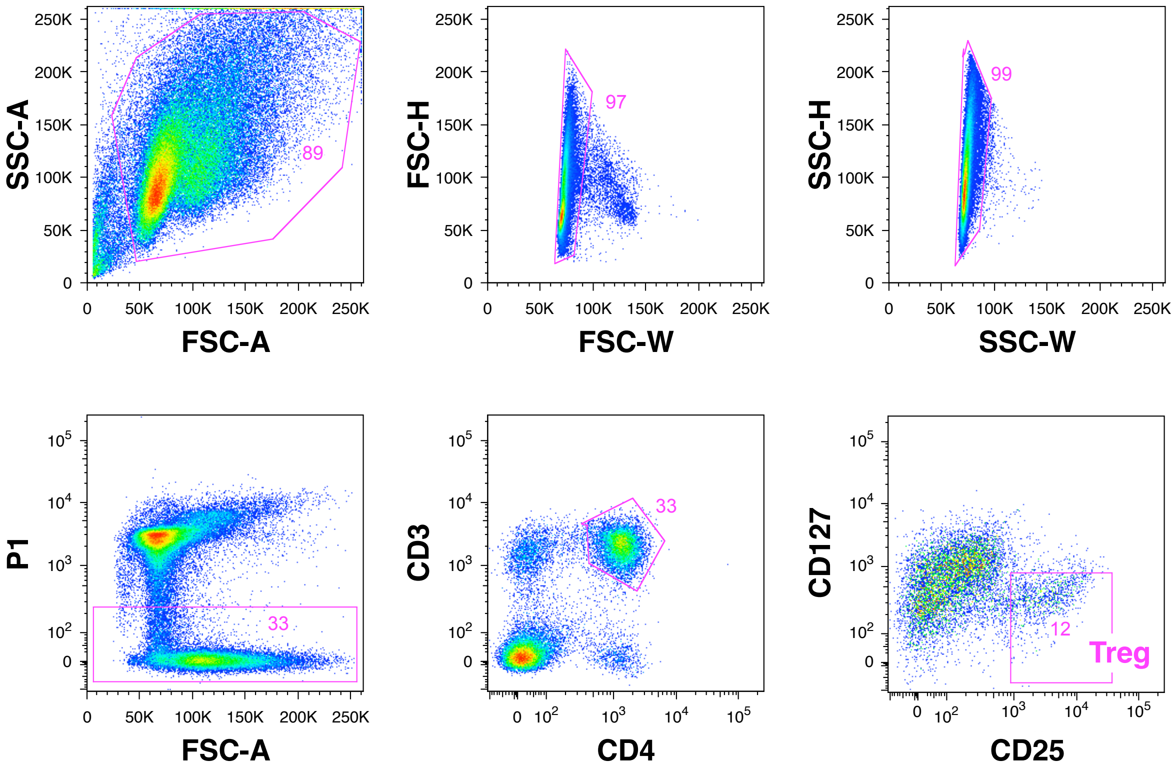

B

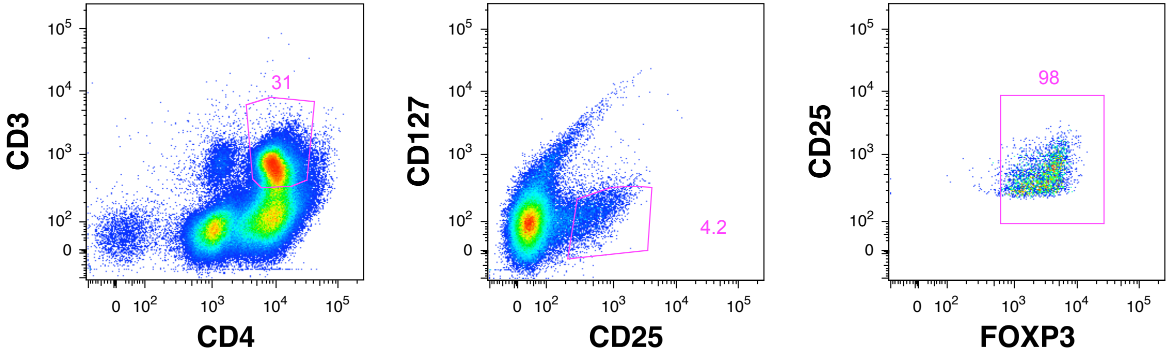

Figure D. Gating strategy and FOXP3 staining. A. Gating strategy used for sort. B. FOXP3 staining.

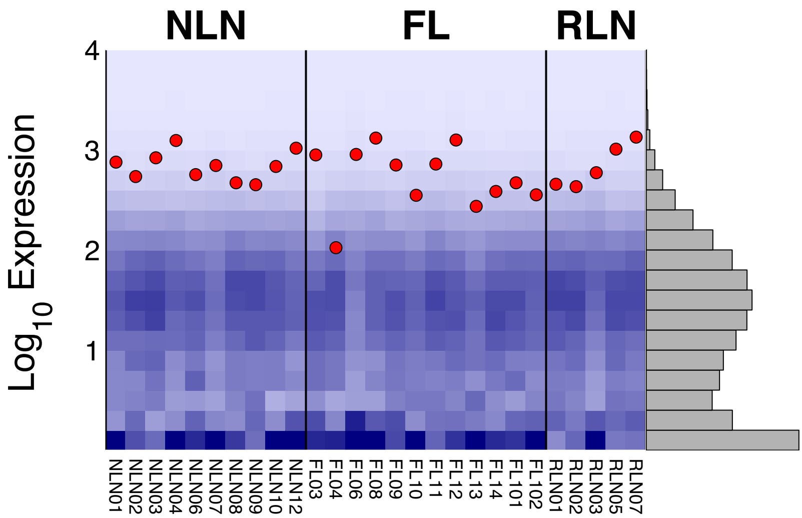

Figure E. FOXP3 expression for all samples. Red circles show log FOXP3 expression level across all 27 samples in RPM. The expression level distribution for all 14725 genes is shown per sample as a heat map, with darker shades of blue indicating larger numbers of genes in the corresponding binned expression range. The average signal distribution is shown to the right as a gray histogram. Note that even for sample FL04 where FOXP3 expression was unusually low relative to the others, its expression was still greater than the peak of the general expression distribution which is around 1.5.

FL vs. NLN

GENE	-log(p)	FDR	Fold
CH25H	5.59	0.001	13.89
MYO7A	6.99	0.000	13.55
CADM1	5.84	0.001	9.88
RCAN2	6.72	0.000	7.74
SMAD1	6.87	0.000	7.00
RGS1	6.67	0.000	6.76
B3GAT1	5.39	0.001	6.31
MAGEH1	6.32	0.000	4.85
TSC22D3	5.27	0.002	4.33
TNFAIP3	5.96	0.001	4.28
CREM	5.86	0.001	4.16
TOX2	5.71	0.001	4.13
NAB1	6.83	0.000	4.03
CLTCL1	5.44	0.001	3.50
SAT1	6.91	0.000	3.34
NFKBIA	5.87	0.001	3.32
JUNB	5.34	0.001	3.14
TSPYL2	5.74	0.001	3.02
SPATS2L	5.27	0.002	2.52
IL6ST	6.12	0.000	2.36
MAF	5.83	0.001	2.16
TRPS1	5.45	0.001	2.04
BTG1	6.25	0.000	1.75
PHACTR2	5.74	0.001	1.60
AKAP13	5.52	0.001	1.57
LDHB	5.95	0.001	-2.05
FAM65B	6.79	0.000	-2.44
LEF1	5.67	0.001	-2.59
TTC39C	7.03	0.000	-2.70
SELL	9.10	0.000	-2.84
CAPN2	6.00	0.001	-2.89
ITGA6	5.44	0.001	-3.12
GBP1	6.61	0.000	-3.16
PRKCA	5.47	0.001	-3.16
CDC25B	5.86	0.001	-3.42
S100A10	6.46	0.000	-3.53
CCR7	6.39	0.000	-4.31
GTSE1	5.28	0.002	-5.06
MGAT4A	5.33	0.001	-5.16
ANXA2	6.84	0.000	-5.39
ANXA2P2	5.61	0.001	-5.40
PKHD1	6.60	0.000	-5.97
LRRN3	6.25	0.000	-6.45
RORC	6.33	0.000	-7.32
LGALS1	5.66	0.001	-8.23
CASP10	5.66	0.001	-9.27
S1PR1	6.83	0.000	-10.28
PLAC8	6.96	0.000	-11.03
CSGALNACT1	7.33	0.000	-11.34
RASGRP4	6.32	0.000	-11.48

RLN vs. NLN

GENE	-log(p)	FDR	Fold
CCL3	4.45	0.055	16.60
ATP9A	4.29	0.057	14.18
MERTK	4.49	0.055	9.16
PLEK	4.16	0.057	5.42
HIST1H2BB	3.84	0.068	5.24
NCRNA00167	4.10	0.057	5.13
FOS	4.09	0.057	4.55
FKBP14	4.19	0.057	4.16
LOC730227	3.98	0.065	4.02
FLJ13224	4.04	0.061	3.45
HIST1H2AK	3.83	0.068	3.17
POU2AF1	4.15	0.057	3.08
RND1	4.23	0.057	2.97
LOC340515	4.53	0.055	2.85
CD69	4.37	0.057	2.68
ZFAND5	3.82	0.068	2.50
PHLDA1	3.75	0.068	2.48
DUSP1	5.25	0.055	2.40
ARRDC3	4.92	0.055	2.25
LOC541471	3.91	0.065	2.07
EIF5	3.83	0.068	2.03
PPP1R15B	3.76	0.068	2.03
SRSF3	4.43	0.055	1.76
PRND	3.91	0.065	1.67
SDHD	4.09	0.057	1.66
SEPN1	3.62	0.068	-1.46
ARHGEF6	4.75	0.055	-1.47
SEPT6	3.66	0.068	-1.48
RBM33	3.55	0.068	-1.50
SPTAN1	3.92	0.065	-1.51
OPTN	3.66	0.068	-1.55
DOCK10	3.92	0.065	-1.58
AKNA	3.53	0.068	-1.59
SP2	3.55	0.068	-1.61
FAM102A	3.52	0.068	-1.63
MLL2	3.51	0.068	-1.69
IL16	3.55	0.068	-1.71
ATP8B2	3.60	0.068	-1.85
SKI	3.50	0.068	-1.89
LFNG	3.85	0.068	-1.92
RORA	4.14	0.057	-1.95
FOXP1	4.43	0.055	-2.01
AAK1	3.95	0.065	-2.05
ATXN7L1	3.52	0.068	-2.10
S1PR1	3.52	0.068	-2.13
PAG1	4.14	0.057	-2.19
KLHDC5	3.58	0.068	-2.30
C1orf56	3.48	0.070	-2.43
BCL9L	4.50	0.055	-2.47
CSGALNACT1	4.52	0.055	-6.46

RLN vs. FL

GENE	-log(p)	FDR	Fold
HIST1H3G	4.40	0.025	9.04
HIST1H3B	5.24	0.025	6.83
TWSG1	4.27	0.025	6.00
CCNA2	4.82	0.025	5.82
BUB1B	4.62	0.025	5.72
HIST1H3F	4.29	0.025	5.69
NLN	5.38	0.025	5.65
CDK1	4.86	0.025	5.45
ESCO2	4.45	0.025	5.28
HIST1H2AI	4.61	0.025	5.17
HIST1H3C	4.33	0.025	5.10
HIST1H4H	4.41	0.025	3.90
ZNF296	4.54	0.025	3.61
PBK	4.30	0.025	3.45
TUBA1B	4.45	0.025	3.20
DUT	4.76	0.025	2.92
CDKN3	4.47	0.025	2.86
CSE1L	4.60	0.025	2.46
SLC25A5	4.75	0.025	2.42
CENPN	4.48	0.025	2.20
LY6E	4.48	0.025	2.15
RPLP0	4.81	0.025	2.12
COX16	4.48	0.025	1.82
PSMB1	4.25	0.025	1.71
RPS24	5.52	0.025	1.56
IK	3.31	0.040	-1.27
BAZ2A	3.19	0.044	-1.40
EML4	3.25	0.042	-1.43
DCAF5	3.00	0.051	-1.51
MXD4	3.06	0.048	-1.53
TULP4	2.99	0.052	-1.57
BRPF3	3.42	0.037	-1.58
MLLT6	3.61	0.031	-1.65
N4BP1	3.08	0.048	-1.67
FAM102A	3.70	0.030	-1.72
CREBBP	3.41	0.037	-1.75
AKAP13	4.64	0.025	-1.77
SPOCK2	4.54	0.025	-1.78
RALGDS	3.85	0.027	-1.86
MEF2D	3.33	0.039	-1.87
CRMP1	3.14	0.045	-1.88
IFFO2	3.18	0.044	-1.90
TSPYL2	3.33	0.039	-2.01
NAB1	3.93	0.025	-2.08
MED15	2.95	0.052	-2.16
PBXIP1	4.56	0.025	-2.19
ZDBF2	2.98	0.052	-2.37
ZFYVE28	2.93	0.054	-2.54
PFKFB3	3.76	0.028	-2.74
JMY	3.80	0.028	-2.93

Table A. Top 25 up- and down-regulated genes based on p-value for all three comparisons. Direction of change is taken with respect to the second group in a comparison (e.g. in FL – NLN, direction is relative to NLN). Fold changes were computed based on the mean log2 expression of each group. Top, down-regulated. Bottom, up-regulated.

	PATHWAY	-log(p) BH	GENES
**FL vs. NLN**	EIF2 Signaling	6.50	*MAPK1, RPL34, RPL27, RPS2, EIF1, RPS13, RPL23A, RPS21, EIF3E, RPS7, RPS29, RPL18A, EIF5, RPL10, RPL32, RPL36, RPL38, RPL18, RPL31, RPSA, RPS27A, RPS24*
	Role of JAK family kinases in IL-6-type Cytokine Signaling	4.54	*IL6ST, SOCS1, SOCS3, MAPK1, MAPK10, STAT3, JAK2, MAPK11*
	Regulation of eIF4 and p70S6K Signaling	3.22	*ITGB1, EIF4EBP2, MAPK1, EIF1, RPS2, ITGA5, RPS13, RPS21, EIF3E, MAPK11, RPS29, RPS7, RPS24, RPSA, RPS27A*
	Growth Hormone Signaling	3.02	*SOCS1, SOCS3, FOS, RPS6KA6, MAPK1, IGF1R, PRKCH, STAT3, JAK2, PRKCA*
	Protein Kinase A Signaling	3.02	*MAPK1, NFATC3, DUSP6, PTPN14, DUSP2, CDKN3, CDC25B, NFKBIA, FLNA, TDP2, DUSP7, GNA13, PRKCA, HIST1H1E, PDE4D, TTN, PTP4A1, AKAP13, H3F3A/H3F3B, DUSP1, CREM, KDELR3, PRKCH, DUSP4, LEF1*
	Prolactin Signaling	3.02	*MYC, SOCS1, SOCS3, FOS, MAPK1, PRKCH, STAT3, JAK2, NR3C1, PRKCA*
	STAT3 Pathway	3.02	*MYC, SOCS1, SOCS3, MAPK1, FLT1, IGF1R, MAPK10, STAT3, JAK2, MAPK11*
	Virus Entry via Endocytic Pathways	3.02	*ITGB1, CD55, FLNA, CLTCL1, HLA-B, ITGA6, ITGA5, PRKCH, ACTG1, ITGB7, PRKCA*
**RLN vs. FL**	Cell Cycle: G2/M DNA Damage Checkpoint Regulation	7.02	*PRKDC, CKS2, YWHAE, CKS1B, TOP2A, CCNB2, PLK1, BRCA1, CDK1, CCNB1*
	Pyrimidine Deoxyribonucleotides De Novo Biosynthesis I	5.85	*TYMS, DUT, NME4, CMPK2, RRM2, NME2, RRM1*
	Estrogen-mediated S-phase Entry	5.57	*MYC, CCNA2, CCNE2, CCNE1, TFDP1, RBL1, CDK1*
	GADD45 Signaling	5.02	*PCNA, CCNE2, CCNE1, BRCA1, CDK1, CCNB1*
	DNA damage-induced 14-3-3σ Signaling	5.02	*CCNE2, CCNE1, CCNB2, BRCA1, CDK1, CCNB1*
	Mitotic Roles of Polo-Like Kinase	5.02	*PLK4, CDC20, PRC1, CCNB2, FBXO5, PLK1, CDK1, CDC27, CCNB1*

Table B. IPA enriched canonical (curated) pathways for selected gene lists. Eight and six pathways were enriched where the Benjamini-Hochberg corrected p-value was < 0.001 for the FL vs. NLN and RLN vs. FL lists, respectively. No pathways for the RLN vs. NLN selected gene list met this criteria.

	qPCR		RNAseq		Flow		qPCR vs. RNAseq	
Gene[§]	p-Value*	FL vs. NLN	FDR[†]	FL vs. NLN	p-Value[‡]	FL vs. NLN	Both Significant	Same Direction
BCL6	0.0330	↑	0.0544	↑	0.0000	↑	Y	Y
CCR7	0.0026	↓	0.0004	↓	0.0001	↓	Y	Y
CXCR4	0.0043	↑	0.0049	↑			Y	Y
S1PR1	0.0260	↓	0.0002	↓			Y	Y
SELL	0.0001	↓	0.0000	↓			Y	Y
CCR6	0.1900	↓	0.0073	↓	0.0355	↓	N	Y
CCL20	0.1800	↓	0.0245	↓			N	Y
CCL3	0.1100	↑	0.0119	↑	0.0524	–	N	Y
IL10	0.0690	↑	0.0834	↑	0.0029	↓	N	Y
KLF2	0.2400	↓	0.0172	↓			N	Y
FOXP3	0.6400	–	0.8975	–			Y	n/a

* Wilcoxon Rank Sum test on target concentrations ($2^{-\Delta\Delta Ct}$) with permutations to compute p-values.

[†] FDR based on RPKM values. See Methods.

[‡] Wilcoxon Rank Sum test on flow cytometry frequencies; permutations to compute p-values.

[§] no ΔΔCt data available for CCL4, CXCL13, CXCR5 (no calibrating sample data for these genes).

Table C. qPCR validation of selected genes. Results of statistical comparisons between FL and NLN samples are shown. qPCR and RNAseq comparisons are based on the same set of samples. RNAseq and flow comparisons only have four NLN samples in common.

ID	FL Grade	Age	Gender	RNAseq & qPCR	Flow	Luminex	Migration	S1P1 qPCR
FL03	1	56	M	■				
FL04	2	48	F	■				
FL06	1	64	F	■				
FL08	2	69	M	■				
FL09	1	59	M	■				
FL10	3A	59	M	■				
FL11	1	53	M	■				
FL12	1	57	M	■				
FL13	1/3	43	M	■				
FL14	1/3	65	M	■				
FL101	3A	50	M	■				
FL102	1	46	F	■				
FL25	3A	-	-		■			
FL26	1	73	M		■			
FL27	1	67	M		■			
FL28	3B	55	M		■			
FL15	2	57	M			■		
FL21	1/2	74	M			■		
FL19	3B	70	M		■	■		
FL20	1	79	M		■	■		
FL22	1	28	M		■	■	■	
FL23	1	-	-			■	■	
FL24	3A	61	M			■	■	■
FL16	1	68	M		■	■	■	■
FL17	1	-	-		■	■	■	■
FL18	1	61	M		■	■	■	■
				12	[illegible]	10	6	4

Table D. Patient characteristics and assays performed on FL samples. For RNAseq, qPCR was performed on selected genes for validation. Numbers at the bottom are the total number of samples per assay.
