# Supplementary material for: Follicular Lymphoma Tregs Have a Distinct Transcription Profile Impacting Their Migration and Retention in the Malignant Lymph Node
Source: PLoS One. 2016 May 26;11(5):e0155347. doi: 10.1371/journal.pone.0155347 (PMC4882026; doi:10.1371/journal.pone.0155347)
Supplement: S2 File — File containing data for flow cytometry, Luminex, migration and S1P1 qPCR assays. (PDF) [file pone.0155347.s002.pdf]

Supporting Data for Nedelkovska et al.

Data for Figure 4:

Protein Expression by Flow (frequencies)												
**GROUP**	**CTLA4 (CD152)**	**CCR7 (CD197)**	**TNFRSF18 (GITR)**	**BCL6**	**CCR6**	**CXCR5**	**RORC**	**CCL3 (MIP-1a)**	**CCL4 (MIP-1b)**	**CXCL13**	**IL10**	**IL16**
FL	87.4	7.7	75.9	11.4	9.7	68.4	15.9	6.1	11.2	0.0	10.2	0.0
FL	89.1	12.8	42.3	10.8	16.4	66.6	6.9	9.0	2.4	1.3	1.0	0.8
FL	82.8	8.8	34.6	8.1	16.3	52.4	2.4	7.5	1.5	1.3	1.3	0.0
FL	84.9	5.0	25.3	13.7	8.7	66.1	4.2	6.6	0.6	0.7	0.6	0.2
FL	89.0	22.9	25.2	13.8	8.8	68.5	11.5	5.9	3.1	2.7	2.0	0.2
FL	97.4	7.1	81.4	9.7	16.0	75.9	8.6	16.1	2.3	1.2	1.1	0.1
FL	80.1	4.2	32.9	11.1	6.6	75.2	5.4	6.2	0.7	0.8	0.7	0.3
FL	85.6	27.2	65.4	11.0	15.1	41.4	13.8	12.0	4.4	7.1	2.2	0.0
FL	89.1	9.1	49.9	12.7	10.3	61.0	9.1	8.5	3.2	3.2	4.0	0.8
FL	93.5	2.6	75.8	15.2	10.7	93.6	5.2	8.4	3.2	1.5	1.4	1.1
NLN	80.1	38.7	38.9	4.1	10.9	19.0	40.0	5.6	2.4	0.0	6.5	0.0
NLN	63.8	40.0	14.1	3.5	12.8	42.5	27.0	6.4	1.5	0.9	5.1	0.3
NLN	63.8	26.2	17.5	4.2	13.7	50.8	23.6	7.6	1.8	1.7	2.5	0.4
NLN	78.9	31.4	23.9	3.1	16.6	25.3	12.0	5.1	1.6	1.0	4.5	0.8
NLN	74.9	35.7	17.2	7.1	9.9	28.3	35.9	4.8	0.5	0.5	3.7	0.4
NLN	82.2	18.5	21.1	5.6	34.6	20.8	29.8	7.2	2.3	2.3	11.7	1.0
NLN	74.0	34.2	17.6	2.8	21.2	43.5	18.2	8.1	2.2	1.8	6.1	1.0
NLN	62.6	37.7	11.2	3.5	12.9	58.1	14.2	4.7	0.9	0.6	3.4	0.2
NLN	74.3	39.4	10.2	3.2	18.4	47.3	10.3	3.6	2.0	1.0	6.8	0.0
NLN	79.7	33.2	24.9	3.3	23.3	45.8	12.6	8.7	3.1	2.1	6.9	2.1

Data for Figure 5:

Cytokine, Chemokine Concentrations (pg/ml)					
**GROUP**	**IL-10**	**IL-16**	**MIP-1α (CCL3)**	**MIP-1β (CCL4)**	**CXCL13**
FL	7.42	97.05	1.42	16.79	6.78
FL	4.12	100.90	*	4.39	0.00
FL	4.04	174.50	*	2.63	0.54
FL	12.16	27.25	*	2.91	0.54
FL	2.57	76.40	0.78	8.20	4.23
FL	10.10	118.50	1.30	6.86	1.64
FL	20.80	88.60	1.40	8.73	0.85
FL	11.89	105.00	*	2.07	4.15
FL	9.56	50.60	ND	4.04	3.01
FL	12.07	24.90	ND	6.83	0.92
NLN	2.31	46.60	ND	ND	0.00
NLN	3.86	27.60	ND	0.63	0.00
NLN	3.51	16.30	ND	0.53	0.00
NLN	13.58	14.60	ND	1.12	0.00
NLN	13.05	27.80	ND	0.31	0.00

ND = values < reliable lower assay limit (RLAL) for both replicates; * = values where one replicate < RLAL. These values were plotted as zero.

Data for Figure 6:

% Migration Normalized to Baseline			
**GROUP**	**S1P**	**CXCL13**	**SDF1**
FL	-4	23	28
FL	0	21	45
FL	-4	15	35
FL	-4	11	65
FL	0	NaN	52
FL	5	23	61
NLN	5	8	51
NLN	4	18	42
NLN	4	6	47
NLN	4	NaN	69
NLN	2	10	55
NLN	8	7	45

Data for Figure C in S1 File:

S1P1 qRT-PCR: Avg ΔCt (3 replicates)			
**GROUP**	**0h**	**24h**	**48h**
FL	1.0872	0.9398	1.0393
FL	1.0073	0.9421	0.9197
FL	1.0183	0.9305	0.9377
FL	0.9879	1.1079	1.0386
